# Monoclonal antibody targeting complement C9 binding domain of *Trichinella spiralis* paramyosin impairs the viability of *Trichinella* infective larvae in the presence of complement

**DOI:** 10.1186/1756-3305-7-313

**Published:** 2014-07-04

**Authors:** Yuwan Hao, Xi Zhao, Jing Yang, Yuan Gu, Ran Sun, Xinping Zhu

**Affiliations:** 1 Department of Parasitology, School of Basic Medical Sciences, Capital Medical University, Beijing, China

**Keywords:** Trichinella spiralis, Immune evasion, Paramyosin, Monoclonal antibody

## Abstract

**Background:**

*Trichinella spiralis* expresses paramyosin (*Ts*-Pmy) not only as a structural protein but also as an immunomodulator that inhibits host complement as a survival strategy. Previous studies demonstrated that *Ts*-Pmy bound to complement components C8 and C9 and inhibited the polymerization of C9 during the formation of the membrane attack complex (MAC). The C9 binding domain of *Ts*-Pmy was identified within 14 amino acid residues at the C-terminus of *Ts*-Pmy. The production of a monoclonal antibody that specifically targets the C9 binding site is necessary for further studies of *Ts*-Pmy function and may be used as a therapeutic agent for *T. spiralis* infection.

**Methods:**

In this study, a monoclonal antibody against the complement C9 binding domain of *Ts*-Pmy (mAb 9G3) was produced using hybridoma technology. The binding activity of the mAb produced for recombinant or native *Ts*-Pmy and the blockade of *Ts*-Pmy binding to C9 by the mAb were assessed by Western blot analysis. The effect of the mAb on the viability of *T. spiralis* was observed by co-incubation of *T. spiralis* with mAb 9G3 in the presence of complement *in vitro* and by passive transfer of the mAb into naive mice following *T. spiralis* larval challenge.

**Results:**

mAb 9G3 was successfully produced against the C9 binding domain of *Ts*-Pmy and bound specifically not only to recombinant *Ts*-Pmy but also to native *Ts*-Pmy expressed in different stages of *T. spiralis*, including adult worms, newborn larvae and muscle larvae. The binding of mAb 9G3 to *Ts*-Pmy efficiently blocked the binding of *Ts*-Pmy to human complement C9, resulting in a significant increase in the complement-mediated killing of newborn larvae *in vitro* and reduced infectivity of *T. spiralis* larvae in mice passively transferred with the mAb.

**Conclusions:**

mAb 9G3 is a specific antibody that binds to the C9 binding domain of *Ts*-Pmy and interferes with *Ts*-Pmy’s complement-binding activity. Therefore, this mAb is a protective antibody that has potential as a preventive and therapeutic agent for *T. spiralis* infection.

## Background

*Trichinella spiralis* is a parasitic nematode that infects humans and other mammals around the world [1]. Trichinellosis, caused by the consumption of raw or undercooked meat contaminated with infective muscle larvae, remains an important infectious disease on a global scale [2]. Due to the predominantly zoonotic importance of infection, new regulations for meat inspection and efficient quality control measures have been studied and enacted in recent years [3]. In addition, the identification of potential vaccine candidates, proteins and protective antibodies has been used as an important strategy for the control of *T. spiralis* infection [4,5].

The host complement system is the first line of defense against pathogenic organisms [6]. Blocking the assembly of complement is a pathogens principal mechanism for escaping from host immune attack [7]. Parasitic nematodes have been suggested to produce compounds capable of inhibiting the assembly and polymerization of the membrane complex attack, thus preventing complement-mediated damage [8]. Subsequent studies revealed that *T. spiralis* worms could bind to complement components [8-10], suggesting that *T. spiralis* contains proteins that bind to and potentially inhibit complement activation to protect against host complement attack.

Paramyosin, which serves as an essential muscle protein in invertebrates, forms the core of thick myofilaments, which determine the length and stability of muscles [11]. In addition to being a structural protein, paramyosin has been defined as a potential vaccine candidate against some helminthiases [12-15]. Additional evidence demonstrated that paramyosin played an important role as an immunomodulatory protein in helminth infections [12,14,16]. Paramyosin, which acts as a complement inhibitor, is capable of inhibiting complement activation by binding to at least three complement components: C1q [17], C8, and C9 [18-20]. In our previous study, a full-length cDNA encoding *T. spiralis* paramyosin (*Ts*-Pmy) was cloned and partial protection against *T. spiralis* infection was achieved in mice by immunizing with recombinant *Ts*-Pmy (r*Ts*-Pmy) [16,21]. In addition, paramyosin expressed on the outer membrane of *T. spiralis* plays an important role in host immunomodulation, specifically by binding to human complement components C8 and C9 and inhibiting the formation of the complement membrane attack complex (MAC), thus creating an effective strategy via which the *Trichinella* parasite can evade host complement attack [20]. Our recent results further identified the exact C9 binding site in *Ts*-Pmy, which was narrowed to 14 amino acid residues within the C-terminus of *Ts*-Pmy between ^866^Val and ^879^Met via fragmental expression and synthesized peptide screening [20]. In the presence of the synthesized C9-binding peptide, human C9 polymerization and human complement-mediated hemolytic activity were inhibited [22].

The immune escape function of paramyosin is an effective survival strategy that allows *T. spiralis* to live within its host. Blocking the complement inhibitory activity of paramyosin could be explored as an alternative strategy for the control of *T. spiralis* infection. Monoclonal antibodies (mAbs) targeting the complement C9 binding site of *T. spiralis* paramyosin were produced and characterized in this study. The viability of *T. spiralis* newborn larvae (NBL) treated with one of these mAbs (mAb 9G3) was impaired in the presence of human serum, and partial protection against *T. spiralis* larval challenge was achieved in mice passively transferred with the mAb against the *Ts*-Pmy C9 binding domain.

## Methods

### Experimental animals

All experimental animals were purchased from the Laboratory Animal Services Center of Capital Medical University (Beijing, China). All experimental procedures were reviewed and approved by the Capital Medical University Animal Care and Use Committee and were consistent with the NIH Guide for the Care and Use of Laboratory Animals.

### Parasites and antigen preparation

*T. spiralis* (ISS 533) was maintained in female ICR mice. Muscle larvae were recovered from infected mice using the standard pepsin digestion method, as described previously [23]. Adult worms were collected from intestines of mice 5–7 days after experimental infection. NBL were obtained from fertile female adult worms cultured overnight in RPMI 1640 containing antibiotics (100 U/ml penicillin and 100 mg/ml streptomycin) at 37°C in the presence of 5% CO_2_. Crude somatic extracts of adult worms, muscle larvae (ML) and NBL were prepared by homogenizing the parasites in PBS, pH 7.4, protein concentrations of the extract supernatants were determined using the BCA assay (Pierce, USA).

### Synthesis of *Ts*-Pmy C9 binding domain peptide

The C9 binding domain in *Ts*-Pmy was identified in our previous study [20], and the binding peptide P25 (KHRSSVSMGKSLSSKVYVMEEGHEY, *Ts*-Pmy861-885), which contains the sequence of the C9 binding site (VSMGKSLSSKVYVM, *Ts*-Pmy866-879), was synthesized using the solid-phase peptide synthesis method, purified up to 95% via preparative RP-HPLC and verified by mass spectrometry (Aviva Bio, China).

### Production of monoclonal antibodies

To increase the immunogenicity of peptide P25, BSA was used as a carrier. Monoclonal antibodies (mAbs) against peptide P25 were produced using hybridoma technology [24]. Briefly, female BALB/c mice (6–8 weeks old) were immunized subcutaneously with 100 μg of BSA conjugated-P25 emulsified with an equal volume of Freund’s complete adjuvant, and boosted twice at 2-week intervals with the same amount of BSA-conjugated peptide emulsified with an equal volume of Freund’s incomplete adjuvant. Three days after the final boost, the mice were sacrificed and the spleen cells were removed and fused with SP2/0 cells at a ratio of 5:1 with 50% (w/v) pre-warmed (37°C) PEG2000 (Sigma–Aldrich, USA). The hybridoma supernatants were screened for antibody activity via ELISA using synthesized peptide P25 as the antigen. The hybridomas secreting antibodies were cloned by limiting dilution. The hybridoma cell clone 9G3, which exhibited stable growth and secreted a high titer of antibody, was selected for further analysis and for the production of ascites in the peritoneal cavities of BALB/c mice.

The mAb-containing culture supernatant and ascitic fluid were collected from BALB/c mice, and the antibody titer was determined using ELISA. The mAbs were purified by affinity chromatography using a Protein A sepharose 4 FF column (GE Healthcare, USA). The subclass of the mAb was determined using a Mouse Monoclonal Antibody Isotyping Kit (Gibco-BRL, USA).

### Immunological recognition of mAb 9G3

To determine whether mAb 9G3 enables the recognition of recombinant or native *Ts*-Pmy, recombinant r*Ts*-Pmy was expressed in *E. coli* BL21 as described previously [20]. Purified r*Ts*-Pmy (0.5 μg), native *Ts*-Pmy containing crude somatic extracts of ML, NBL or adult worms (5 μg each) and recombinant heat shock protein-70 of *T. spiralis* (*Ts*-Hsp70) [25] (1 μg), which served as a non-relevant recombinant protein control, were separated via 12% SDS-PAGE under reducing conditions, and transferred onto a nitrocellulose (NC) membrane (Millipore, USA). After blocking with 5% milk in PBS for 1 h at room temperature, the membrane was incubated with mAb 9G3 (0.2 μg/ml) in 1% skimmed milk–PBS for 1 h at room temperature. IRDye 800CW-labeled goat anti-mouse IgG (LI-COR, Germany) was used as the secondary antibody. Recognition was detected and imaged using the Odyssey infrared imaging system (LI-COR, Germany).

To determine whether mAb 9G3 is able to recognize native *Ts*-Pmy expressed on the surface of the parasite, longitudinal sections of *T. spiralis* ML were prepared. The sections were blocked with normal goat serum (1:10) for 30 min and subsequently incubated with 5 μg/ml of 9G3 in 1× PBS, pH 7.4 with 0.05% Tween-20 (PBST) for 1 h at room temperature. Normal mouse serum was used at a 1:100 dilution as a control. The sections were washed with PBST and subsequently incubated with a 1:200 dilution of an Alexa Fluor 488-labeled goat anti-mouse IgG antibody for 1 h, followed by the addition of the DAPI fluorescent nuclear stain (1.25 μg/ml). The labeling images were obtained via confocal laser scanning microscopy.

### Inhibition of r*Ts*-Pmy binding to C9 by mAb 9G3

To determine whether mAb 9G3 against the C9 binding domain of *Ts*-Pmy is able to inhibit the binding of r*Ts*-Pmy to human complement C9, r*Ts*-Pmy (2 μg) was transferred onto a NC membrane (Millipore, USA) and subsequently incubated with various amounts of mAb 9G3 (0, 2, and 4 μg) for 1 h at room temperature. BSA or mAb 7E2 (10 μg each), which is another monoclonal antibody against r*Ts*-Pmy1-315 that lacks the ability to bind to the complement binding site [26], were used as controls for the binding assay. After washing, the membrane strips were incubated with human C9 (1 μg/ml) (Merck, Germany) at 37°C for 2 h and subsequently incubated with a rabbit anti-C9 polyclonal antibody (0.2 μg/ml) (Abnova, Taiwan) for 1 h at room temperature. IRDye 800CW-conjugated goat anti-rabbit IgG (50 ng/ml) (LI-COR, Germany) was used as the secondary antibody. All membrane strips were detected and imaged using the Odyssey infrared imaging system (LI-COR, Germany).

### mAb 9G3 enhanced complement-mediated killing of NBL *in vitro*

A previous study demonstrated that r*Ts*-Pmy could bind to complement components C8 and C9 and inhibit the complement-mediated killing of NBL [20]. To determine whether blocking the *Ts*-Pmy C9-binding domain with mAb 9G3 would enhance the complement-mediated killing of *T. spiralis* larvae, freshly obtained NBL were pretreated with different amounts of mAb 9G3 (2, 20, or 40 μl of a 1 mg/ml solution) in a final volume of 150 μl/well in a 96-well plate for 30 min at room temperature. The same amount of mAb 7E2 was used as non-relevant antibody control and normal mouse serum was used as negative antibody control. Subsequently, 100 μl of fresh normal human serum was added into each well as a source of complement for an overnight incubation at 37°C in a 5% CO_2_ incubator. Heat-inactivated human serum (30 min at 56°C) served as a control. The mortality of the NBL after the incubation was assessed based on motility under an inverted microscope (worms without any movement during 30 seconds of observation and total stretch-out were scored as dead) [18]. The experiments were performed in triplicate. The percent mortality was calculated in the complement-treated group compared with the heat-inactivated serum group.

### Passive immunization and parasite challenge

To evaluate the possible protective function of mAb 9G3 in the defense against *T. spiralis* infection via blocking the C9-binding site of surface-expressed *Ts*-Pmy, a group of six female BALB/c mice that were six to eight weeks of age underwent passive transfer with 0.5 mg of mAb 9G3 in a total volume of 0.1 ml per mouse 2 h before oral challenge with 500 *T. spiralis* ML. Another boost containing the same amount of mAb 9G3 was administered 4 days after infection. *T. spiralis*-infected sera and normal mouse sera at the same dose were used as the positive and negative controls, respectively. Infected mouse sera were obtained from BALB/c mice 45 days post-infection with 500 *T. spiralis* ML. The reduction of the worm burden was evaluated by counting the number of muscle larvae collected from whole muscle tissue of mice 45 days after the challenge infection [16].

### Statistical analysis

The data were expressed as the mean ± standard error (S.E.). Statistical analysis was performed with GraphPad Prism software (San Diego, USA) using One-Way ANOVA. Differences for which P < 0.05 were considered statistically significant.

## Results

### Production and characterization of mAb 9G3

**Figure 1:**
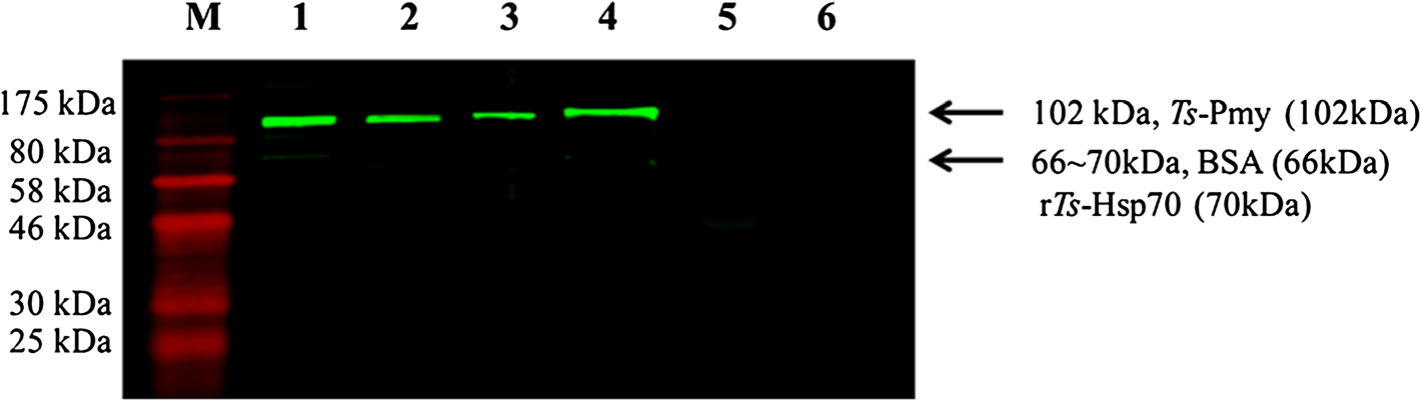
**Recognition of recombinant and native *Ts*-Pmy (~102 kDa) by mAb 9G3, as demonstrated by Western blot.** M, protein marker; Lane 1, adult extracts (5 μg); Lane 2, ML extracts (5 μg); Lane 3, NBL extracts (5 μg); Lane 4, r*Ts*-Pmy with a His-tag (0.5 μg); Lane 5,BSA(1 μg); Lane 6, irrelevant recombinant *Ts*-Hsp70 control (1 μg). After transfer of the proteins to the nitrocellulose membrane, the membrane was incubated with mAb 9G3 (0.2 μg/ml). IRDye 800CW-labeled goat anti-mouse IgG was used as the secondary antibody.

Monoclonal antibodies (mAbs) against the *Ts*-Pmy C9 binding site were made by fusing splenocytes from mice immunized with the C9-binding site peptide (P25) with SP2/0. A total of five positive hybridoma cell lines were cloned by limiting dilution based on their high titers against peptide P25 during ELISA, these cell lines were designated as 5E9, 9G3, 10 F10, 14C10, and 14H9. The subclasses of all of these mAbs were determined to be IgG1. One of these antibodies, mAb 9G3, exhibited the highest absorbance value during ELISA and also exhibited stable growth. Therefore, this clone was selected for further study. The 9G3 hybridoma cells were injected into BALB/c mice for ascitic fluid production. The antibody titers of the cell culture supernatants and the ascitic fluid against P25 were 1:3 200 and 1:516 000, respectively. Western blot analysis revealed that mAb 9G3 recognized not only the full-length recombinant r*Ts*-Pmy protein (~110 kDa) but also the native protein in adult and larval worms of *T. spiralis* (Figure 1).

### Immunolocalization analysis

**Figure 2:**
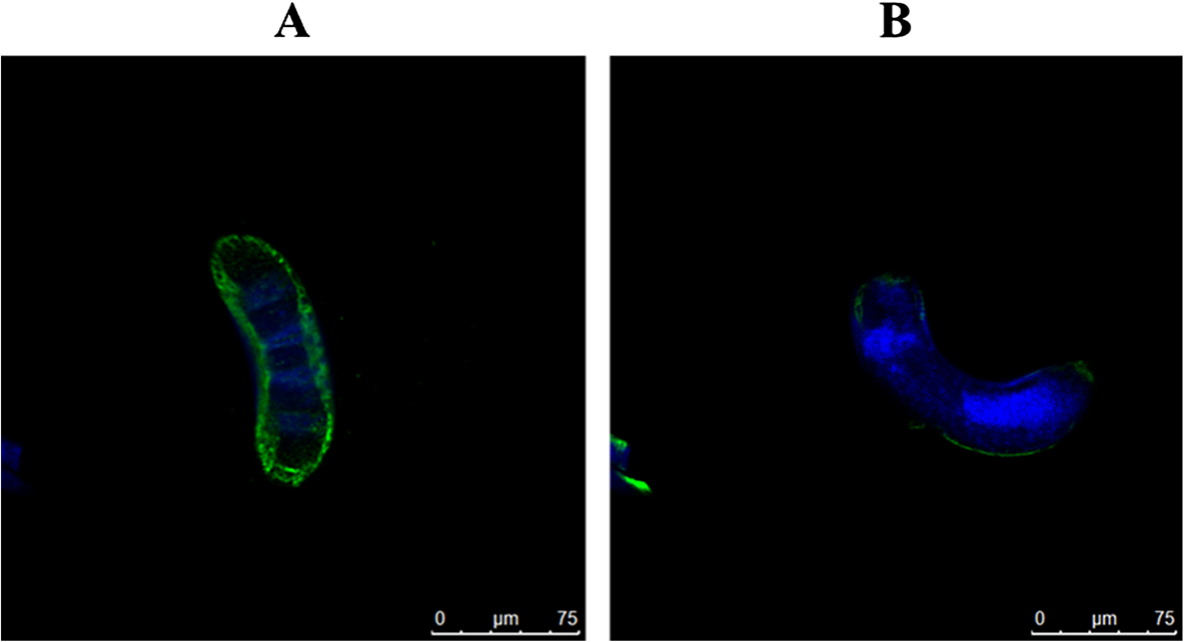
**Immunofluorescent detection of native *Ts*-Pmy expressed on the surface of *T. spiralis *ML using the anti-*Ts *-Pmy C9 binding domain mAb 9G3.** The longitudinal section of ML was incubated with mAb 9G3 (5 μg/ml), and subsequently incubated with Alexa Fluor 488-labeled goat anti-mouse IgG antibody (in green) or DAPI to label nuclei (in blue) **(A)**. Normal mouse serum at the same dilution was used as a negative control **(B)**. The scale bars represent 15 μm.

An immunofluorescence assay demonstrated that the anti-*Ts*-Pmy C9 binding site mAb 9G3 could strongly recognize native *Ts*-Pmy expressed on the surface of ML longitudinal sections (Figure 2). This finding is consistent with our previous study, which demonstrated that *Ts*-Pmy was expressed on the outer membrane of the cuticle of NBL and adult worms [20]. No significant staining was observed using normal mouse serum at the same dilution. All larvae exhibited nuclei that stained with DAPI.

### mAb 9G3 blocks r*Ts*-Pmy binding to C9

**Figure 3:**
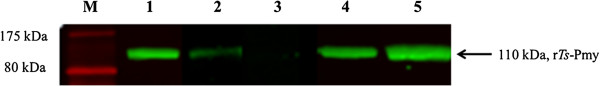
**Inhibition of r *Ts*-Pmy binding to C9 by the addition of different amounts of mAb 9G3, as determined by Western blot.** r*Ts*-Pmy (2 μg) was transferred onto a NC membrane and incubated with various amounts of mAb 9G3. Lane 1: PBS only; Lane 2: mAb 9G3 (2 μg); Lane 3: mAb 9G3 (4 μg). Lane 4: BSA (10 μg) and Lane 5: irrelevant mAb 7E2 control (10 μg). After washing, the membrane was incubated with human C9 (1 μg/ml) at 37°C for 2 h and subsequently incubated with rabbit anti-C9 polyclonal antibody (0.2 μg/ml) for 1 h at room temperature. IRDye 800CW-labeled goat anti-rabbit IgG (50 ng/ml) was used as the secondary antibody.

The binding of r*Ts*-Pmy to C9 was significantly inhibited by mAb 9G3 in a dose dependent manner. When the amount of mAb 9G3 was increased to 4 μg, the antibody nearly completely blocked the binding of r*Ts*-Pmy (2 μg) to C9 (1 μg/ml) (Figure 3). As expected, the negative controls mAb 7E2 and BSA exerted no inhibitory effect on the binding of r*Ts*-Pmy to C9, even at high concentrations (10 μg).

### Enhanced complement-mediated killing of NBL incubated with mAb 9G3

**Figure 4:**
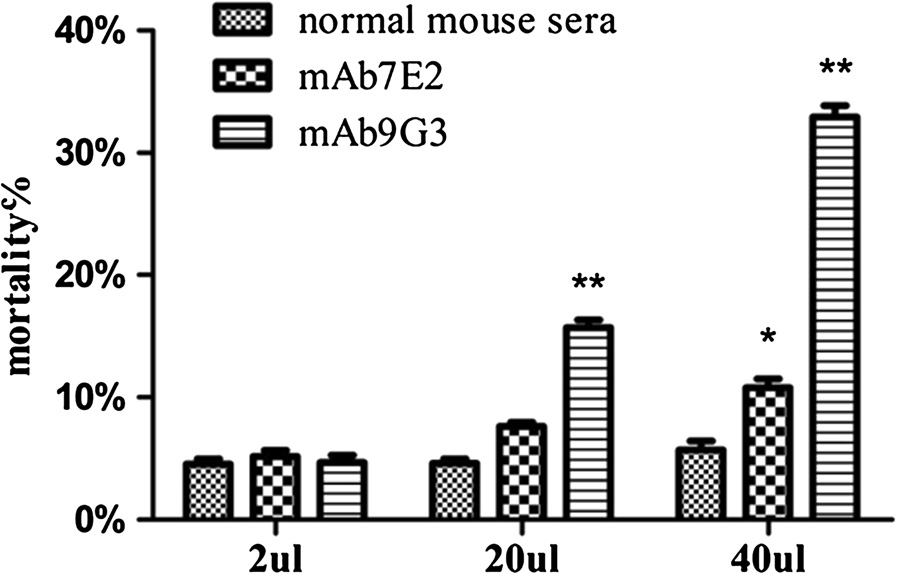
**Enhanced complement-mediated killing of *T. spiralis *NBL incubated with mAb 9G3.** NBL were pretreated with different volumes (2 μl, 20 μl and 40 μl) of mAb 9G3 (1 mg/ml), the irrelevant mAb 7E2 (1 mg/ml) or normal mouse serum prior to the addition of 100 μl of normal human serum. Heat-inactivated human serum served as a control. The mortality of the NBL was assessed under an inverted microscope. The results are presented as the arithmetic mean ± standard error (SE) from triplicate experiments. **p < 0.01, *p < 0.05 compared with the normal mouse sera control.

A previous study demonstrated that *Ts*-Pmy plays an important role in protecting *Trichinella* parasites, particularly NBL, from being attacked by host complement by binding to C8 or C9 [18]. In this study, NBL were incubated with mAb 9G3 to block the binding site of native surface-expressed *Ts*-Pmy for complement C9. The NBL were xthen incubated with fresh human serum as a source of complement. The results demonstrated that the complement-mediated killing of NBL was significantly enhanced by incubation with mAb 9G3 in a dose-dependent manner compared with the normal mouse serum group. When up to 40 μl of a 1 mg/ml solution of mAb 9G3 was added, 32.9% of NBL were killed compared with 4.3% of NBL in the normal mouse sera control group (40 μl added). No significant increases in larval mortality were observed in the group incubated with the monoclonal antibody mAb 7E2 until the amount of mAb 7E2 reached 40 μl of a 1 mg/ml antibody solution, however, the mortality (10.8%) of this treatment was much lower than that caused by the same amount of mAb 9G3 (32.9%) (Figure 4).

### Protection against challenge infection in mice adoptively transferred with mAb 9G3

**Figure 5:**
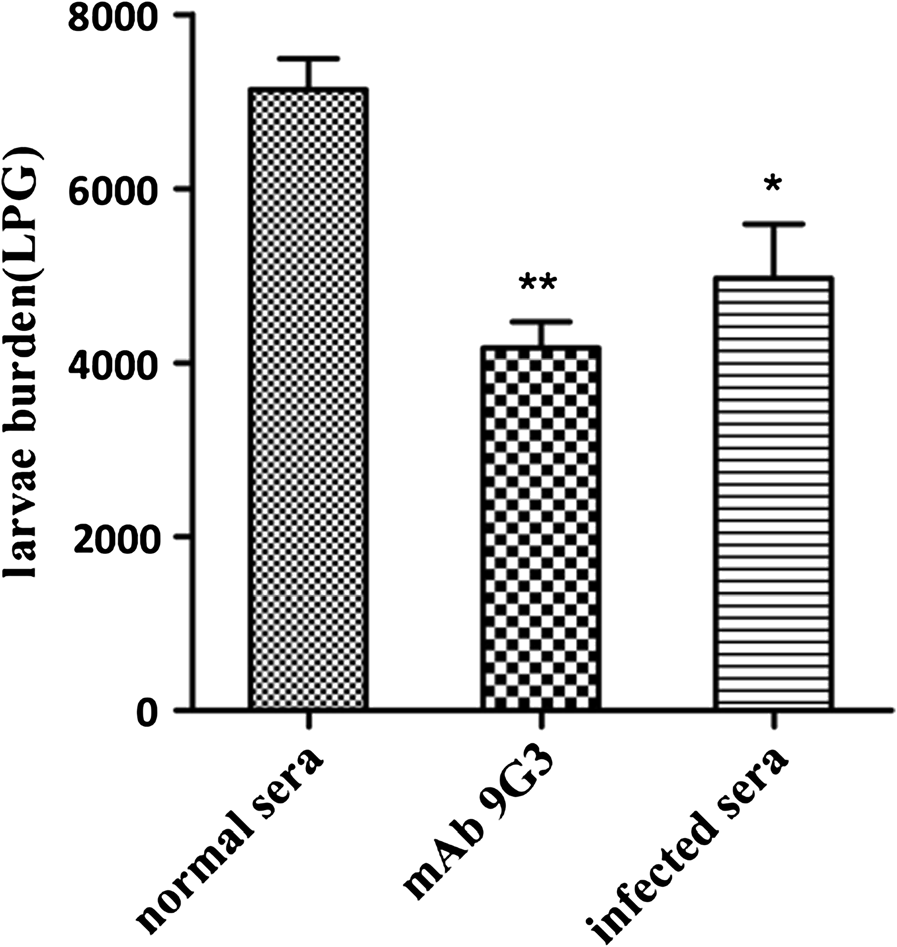
**Protection of mice against *T. spiralis *larval challenge after passive transfer of mAb 9G3 or *T. spiralis*-infected mouse sera.** Larvae were collected from the muscles of BALB/c mice following the passive transfer of mAb 9G3 and a subsequent challenge infection with 500 *T. spiralis* larvae. The results are presented as the arithmetic mean of six mice per group ± standard error (SE). **p < 0.01, *p < 0.05 compared with mice transferred with normal mouse sera.

The challenge results demonstrated that after the passive transfer of 0.5 mg of mAb 9G3 in a total volume of 100 μl to BALB/c mice, each mouse exhibited a 42.6% reduction in muscle larvae burden compared with the normal mouse sera transfer control (*P* < 0.01) (Figure 5). As a positive control, mice that received the same volume of *T. spiralis*-infected mouse sera (100 μl) displayed a 34.3% reduction in muscle larvae burden (*P* < 0.05). The results presented here demonstrate that mAb 9G3 provides protective immunity against *T. spiralis* infection that is similar to or better than that induced by *T. spiralis*-infected sera.

## Discussion

Due to its ability to rapidly recognize and eliminate microorganisms, the complement system is an essential and efficient component of the human innate immune system [6]. During co-evolution with their hosts, parasites developed a multitude of mechanisms for evading attacks by the host immune system. Parasite-produced compounds that bind to and inactivate the host’s complement components have been identified in several parasites as effective immune escape mechanisms [4,6-8,16,17]. Paramyosin is one of the most important molecules produced by helminths to defense from the host’s complement attack [10,12,14]. In particular, *T. spiralis*-produced paramyosin (*Ts*-Pmy) was demonstrated to bind to human complement components C8 and C9 and subsequently inhibit the polymerization of C9 during the formation of the membrane attack complex (MAC), thus protecting *Trichinella* larvae from being attacked by the host complement system [20]. The complement C9 binding domain of *Ts*-Pmy was identified within 14 amino acid residues (^866^Val-^879^Met) at the C-terminus of *Ts*-Pmy [22]. Theoretically, a specific antibody targeting the complement binding domain of *Ts*-Pmy should block the binding of *Ts*-Pmy to human complement, exposing the parasites to attack by the host complement system. Therefore, such an antibody would be a protective antibody against *T.spiralis* infection.

To produce specific antibodies targeting the C9 binding domain of *Ts*-Pmy, a 25 amino-acid peptide (P25) covering *Ts*-Pmy861-885, including the 14 amino-acid sequence of the C9 binding site (VSMGKSLSSKVYVM, Ts-Pmy866-879), was synthesized and used to immunize mice for the production of monoclonal antibodies. Although the C9 binding site in *Ts*-Pmy was narrowed to 14 amino acid residues (^866^Val-^879^Met), the extension of the amino acid sequence on both sides up to 25 amino acids may increase the immunogenicity of the peptide. The production of monoclonal antibodies against synthetic peptides is a versatile tool in the molecular and functional analysis of proteins [27-29]. However, some factors should be considered during the design of peptides for the production of anti-peptide antibodies, including the length of the peptides, the location of the peptides in the native molecule and hydrophilicity of the peptides [30]. In this study, the synthesized P25 peptide was conjugated to BSA to increase its immunogenicity. To exclude the possible production of mAbs against the conjugated BSA, the hybridoma culture supernatants were screened directly against synthesized peptide as an antigen coated on plate during ELISA. Among the five stable hybridoma cell lines, only the stable cell line 9G3 secreted an antibody that recognized not only full-length recombinant *Ts*-Pmy, but also native *Ts*-Pmy in *T. spiralis* adult and larval (ML and NBL) extracts. Immunolocalization on longitudinal sections of ML demonstrated that mAb 9G3 also recognized *Ts*-Pmy expressed on the surface of *Trichinella* larva, which is consistent with our previous study that demonstrated that *Ts*-Pmy was present on the outer membrane of the cuticle of adults and NBL [18]. The universal distribution of *Ts*-Pmy in different developmental stages of *T. spiralis* provides a more adequate experimental basis for the role of paramyosin as a potential modulator of the host immune system during different parasitic stages.

In addition to the specific binding of mAb 9G3 to native *Ts*-Pmy expressed in different developmental stages of *T. spiralis*, this antibody also efficiently inhibits the binding of *Ts*-Pmy to human complement C9, suggesting that this antibody possesses the ability to functionally block the complement binding activity of *Ts*-Pmy. To determine whether blocking the C9-binding domain of *Ts*-Pmy would inhibit the protection employed by *Ts*-Pmy against complement attack and therefore enhance the complement-mediated killing of *T. spiralis* parasites, we incubated *T. spiralis* NBL with mAb 9G3 before adding fresh human serum as a source of complement. NBL were chosen as a target for the complement killing assay because the NBL stage is the stage that migrates through the blood and lymphatic circulation to muscular tissue and is exposed directly to the host immune system, thus, this stage should develop sophisticated abilities to evade complement attack, such as complement-binding *Ts*-Pmy [18], as a survival strategy [5]. Blocking *Ts*-Pmy on the surface of *T. spiralis* NBL by incubating live NBL with mAb 9G3 resulted in significantly enhanced complement-mediated killing of NBL in the presence of fresh human serum *in vitro*, suggesting that blocking the C9-binding domain of *Ts*-Pmy with mAb 9G3 efficiently inhibits the function of *Ts*-Pmy that protects the parasite from being attacked by host complement. After incubation with mAb 7E2, which is a mAb that binds to the N-terminus of *Ts*-Pmy but not to the complement-binding domain of *Ts*-Pmy, *T. spiralis* larvae exhibited similar levels of viability or reduced levels of viability when large amounts of antibody were added, however, the complement-mediated killing of larvae was much lower than that induced by incubation with mAb 9G3 (Figure 4). The limited complement-mediated larval killing by a mAb that does not bind to the complement-binding domain of *Ts*-Pmy (mAb 7E2) may be a result of complement activation through the classic antibody-mediated pathway. The significantly higher complement-mediated larval killing by the anti-C9 binding domain of *Ts*-Pmy mAb than by the mAb that is not related to the complement binding site of *Ts*-Pmy further indicates that *Ts*-Pmy protects *Trichinella* parasites from being attacked by host complement by inhibiting the alternative pathway of complement activation [6]. Impaired infectivity of *T. spiralis* parasites was also observed in mice passively transferred with the anti-C9 binding domain of *Ts*-Pmy mAb 9G3. The protection level induced by mAb 9G3 (42.6%) was higher than that elicited by *T. spiralis* infected mouse sera (34.3%) (Figure 5).

This study further elucidates the mechanism of nematode paramyosin involved in the immunomodulation of host immune response and the specific monoclonal antibody against paramyosin’s functional domain could be used as a therapeutic or preventive agent for Trichinellosis. However, except for the binding site for C9 on *Trichinella*-secreted paramyosin, there may be more binding sites for other components of complement that may also contribute to evading complement attack for the survival of parasite in the host, which is under investigation.

## Conclusions

In summary, in this study, we demonstrated that the monoclonal antibody 9G3 that targets the complement C9 binding domain of *Ts*-Pmy could efficiently block the complement inhibitory activity of *Ts*-Pmy, resulting in a significant increase in the complement-mediated killing of newborn larvae *in vitro* and reduced infectivity of *T. spiralis* larvae in mice passively transferred with the mAb. Therefore, mAb 9G3 is a protective antibody that has potential as a preventive and therapeutic agent for *T. spiralis* infection.

## Competing interests

The authors declare that they have no competing interests.

## Authors’ contributions

YWH performed the experiments and drafted the manuscript. XZ, JY, YG, and RS performed some of the experiments. XPZ designed the study and revised the manuscript. All authors read and approved the final manuscript.

## References

[B1] Gottstein B Pozio E Nockler K Epidemiology, diagnosis, treatment, and control of trichinellosis Clin Microbiol Rev 2009 22 1 127 145 10.1128/CMR.00026-08 19136437 PMC2620635

[B2] Dupouy-Camet J Trichinellosis: a worldwide zoonosis Vet Parasitol 2000 93 3–4 191 200 11099837 10.1016/s0304-4017(00)00341-1

[B3] Dupouy-Camet J Presidential address of ICT12 Conference: “Trichinella and trichinellosis–a never ending story” Vet Parasitol 2009 159 3–4 194 196 19054620 10.1016/j.vetpar.2008.10.064

[B4] Zhang YL Wang ZQ Li LG Cui J Molecular characterization of *Trichinella spiralis* aminopeptidase and its potential as a novel vaccine candidate antigen against trichinellosis in BALB/c mice Parasit Vectors 2013 6 1 246 10.1186/1756-3305-6-246 23972034 PMC3765106

[B5] Zumaquero-Rios JL Garcia-Juarez J De-la-Rosa-Arana JL Marcet R Sarracent-Perez J *Trichinella spiralis*: monoclonal antibody against the muscular larvae for the detection of circulating and fecal antigens in experimentally infected rats Exp Parasitol 2012 132 4 444 449 10.1016/j.exppara.2012.09.016 23026455

[B6] Lambris JD Ricklin D Geisbrecht BV Complement evasion by human pathogens Nat Rev Microbiol 2008 6 2 132 142 10.1038/nrmicro1824 18197169 PMC2814840

[B7] Bruschi F The immune response to the parasitic nematode *Trichinella* and the ways to escape it. From experimental studies to implications for human infection Curr Drug Targets Immune Endocr Metabol Disord 2002 2 3 269 280 10.2174/1568008023340523 12476491

[B8] Hong Y Kim CW Ghebrehiwet B *Trichinella spiralis*: activation of complement by infective larvae, adults, and newborn larvae Exp Parasitol 1992 74 3 290 299 10.1016/0014-4894(92)90152-Z 1582481

[B9] Kennedy MW Kuo YM The surfaces of the parasitic nematodes *Trichinella spiralis* and *Toxocara canis* differ in the binding of post-C3 components of human complement by the alternative pathway Parasite Immunol 1988 10 4 459 463 10.1111/j.1365-3024.1988.tb00235.x 2971916

[B10] Nareaho A Saari S Meri S Sukura A Complement membrane attack complex formation and infectivity of *Trichinella spiralis* and *T. nativa* in rats Vet Parasitol 2009 159 3–4 263 267 19038499 10.1016/j.vetpar.2008.10.037

[B11] Elfvin M Levine RJ Dewey MM Paramyosin in invertebrate muscles. I. Identification and localization J Cell Biol 1976 71 1 261 272 10.1083/jcb.71.1.261 824292 PMC2109740

[B12] Lanar DE Pearce EJ James SL Sher A Identification of paramyosin as schistosome antigen recognized by intradermally vaccinated mice Science 1986 234 4776 593 596 10.1126/science.3094144 3094144

[B13] Nanduri J Kazura JW Paramyosin-enhanced clearance of *Brugia malayi* microfilaremia in mice J Immunol 1989 143 10 3359 3363 2809205

[B14] Muhlschlegel F Sygulla L Frosch P Massetti P Frosch M Paramyosin of *Echinococcus granulosus*: cDNA sequence and characterization of a tegumental antigen Parasitol Res 1993 79 8 660 666 10.1007/BF00932508 8295903

[B15] Ferrer E Moyano E Benitez L Gonzalez LM Bryce D Foster-Cuevas M Davila I Cortez MM Harrison LJ Parkhouse RM Garate T Cloning and characterization of *Taenia saginata* paramyosin cDNA Parasitol Res 2003 91 1 60 67 10.1007/s00436-003-0895-5 12898225

[B16] Yang J Yang Y Gu Y Li Q Wei J Wang S Boireau P Zhu X Identification and characterization of a full-length cDNA encoding paramyosin of *Trichinella spiralis* Biochem Biophys Res Commun 2008 365 3 528 533 10.1016/j.bbrc.2007.11.012 18021743

[B17] Laclette JP Shoemaker CB Richter D Arcos L Pante N Cohen C Bing D Nicholson-Weller A Paramyosin inhibits complement C1 J Immunol 1992 148 1 124 128 1727860

[B18] Deng J Gold D LoVerde PT Fishelson Z Mapping of the complement C9 binding domain in paramyosin of the blood fluke *Schistosoma mansoni* Int J Parasitol 2007 37 1 67 75 10.1016/j.ijpara.2006.09.011 17123534

[B19] Deng J Gold D LoVerde PT Fishelson Z Inhibition of the complement membrane attack complex by *Schistosoma mansoni* paramyosin Infect Immun 2003 71 11 6402 6410 10.1128/IAI.71.11.6402-6410.2003 14573661 PMC219572

[B20] Zhang Z Yang J Wei J Yang Y Chen X Zhao X Gu Y Cui S Zhu X *Trichinella spiralis* paramyosin binds to C8 and C9 and protects the tissue-dwelling nematode from being attacked by host complement PLoS Negl Trop Dis 2011 5 7 e1225 10.1371/journal.pntd.0001225 21750743 PMC3130009

[B21] Yang J Gu Y Yang Y Wei J Wang S Cui S Pan J Li Q Zhu X *Trichinella spiralis*: immune response and protective immunity elicited by recombinant paramyosin formulated with different adjuvants Exp Parasitol 2010 124 4 403 408 10.1016/j.exppara.2009.12.010 20045697

[B22] Zhao X Hao Y Yang J Gu Y Zhu X Mapping of the complement C9 binding domain on *Trichinella spiralis* paramyosin Parasit Vectors 2014 7 1 80 10.1186/1756-3305-7-80 24564979 PMC3937825

[B23] Dennis DT Despommier DD Davis N Infectivity of the newborn larva of *Trichinella spiralis* in the rat J Parasitol 1970 56 5 974 977 10.2307/3277516 5504535

[B24] Yokoyama WM Production of Monoclonal Antibodies Current Protocols in Immunology 1995 2 5 10.1002/0471142735.im0205s74 18432969

[B25] Wang S Zhu X Yang Y Yang J Gu Y Wei J Hao R Boireau P Cui S Molecular cloning and characterization of heat shock protein 70 from *Trichinella spiralis* Acta Trop 2009 110 1 46 51 10.1016/j.actatropica.2009.01.003 19185561

[B26] Wei J Gu Y Yang J Yang Y Wang S Cui S Zhu X Identification and characterization of protective epitope of *Trichinella spiralis* paramyosin Vaccine 2011 29 17 3162 3168 10.1016/j.vaccine.2011.02.072 21382481

[B27] Iankov ID Penheiter AR Carlson SK Galanis E Development of monoclonal antibody-based immunoassays for detection of *Helicobacter pylori* neutrophil-activating protein J Immunol Methods 2012 384 1–2 1 9 22750540 10.1016/j.jim.2012.06.010 PMC3691681

[B28] Maleki LA Majidi J Baradaran B Abdolalizadeh J Akbari AM Production and characterization of murine monoclonal antibody against synthetic peptide of CD34 Hum Antibodies 2013 22 1 1 8 24284303 10.3233/HAB-130265

[B29] Kajiura S Yashiki T Funaoka H Ohkaru Y Nishikura K Kanda T Ajioka Y Igarashi M Hatakeyama K Fujii H Establishment and characterization of monoclonal and polyclonal antibodies against human intestinal fatty acid-binding protein (I-FABP) using synthetic regional peptides and recombinant I-FABP J Immunoassay Immunochem 2008 29 1 19 41 18080878 10.1080/15321810701735005

[B30] Tanaka T Slamon DJ Cline MJ Efficient generation of antibodies to oncoproteins by using synthetic peptide antigens Proc Natl Acad Sci U S A 1985 82 10 3400 3404 10.1073/pnas.82.10.3400 3858828 PMC397783

